# Examining the role of early bilingualism on interference suppression and prefrontal connectivity

**DOI:** 10.3389/fnint.2025.1591250

**Published:** 2025-12-17

**Authors:** Matthew L. Cook, Lisa K. Boyce, Allison S. Hancock, Makenzy S. Turner, Spencer D. Bradshaw

**Affiliations:** 1 Department of Human Development and Family Studies, Utah State University, Logan, UT, United States; 2 Department of Psychology, Utah State University, Logan, UT, United States

**Keywords:** fNIRS, functional connectivity, bilingualism, preschool, inhibitory control

## Abstract

Inhibitory control is a core cognitive function that is primarily associated with activation in the prefrontal cortex (PFC) and is the cognitive function that inhibits impulses, thoughts, and suppresses irrelevant information to an identified goal or task. Prior research suggests that bilingualism may affect brain activity related to inhibitory control, yet few studies have compared functional activity between monolingual and bilingual children. The current study used functional near-infrared spectroscopy (fNIRS) to examine region of interest comparisons and task-state functional connectivity across the PFC during an interference suppression Simon task with 13 bilingual (East Asian or Ibero-romance paired with English) and 13 age-matched English monolingual preschoolers. Results showed no significant differences in behavioral measures of interference suppression. However, bilingual preschoolers showed lower oxygenated hemoglobin activation and more localized patterns of connectivity within the PFC, suggesting more efficient processing during suppression compared to their monolingual peers. This may reflect the bilingual experience of regularly suppressing their second language when not in use, thus facilitating neural efficiency. These findings contribute to the growing body of literature on bilingual cognitive development suggesting that functional connectivity during executive function may differ in bilingual children, even at a young age, despite no observable behavioral differences. This highlights the importance of integrating neuroimaging with behavioral data to gain a more comprehensive understanding of bilingual cognitive development.

## Introduction

Executive Function (EF) refers to a set of cognitive processes that enable goal-directed behavior and are primarily supported by the prefrontal cortex (Jones and Graff-Radford, 2021). Inhibitory control falls under EF and refers to the ability to suppress irrelevant thoughts or actions (Rothbart and Posner, 1985). Inhibitory control undergoes rapid development during early childhood (3–5 years old, Moriguchi, 2014, Zelazo and Carlson, 2012) and is crucial for later academic achievement, emotion regulation, and social success (Anthony and Ogg, 2020; Beisly and Jeon, 2024; Sasser et al., 2015). A longitudinal study further supports the importance of inhibitory control over the lifespan, finding that high self-control skills between ages 3 and 11 resulted in significantly better health, higher incomes, and lower rates of criminal conviction in adulthood (Moffitt et al., 2011).

Bilingualism has been proposed as one factor that may influence the development of EF through neural adaptation. Bialystok (2024) proposes that bilingualism facilitates “adaptation,” where neural networks are modified through experience, potentially enhancing attention skills across multiple domains. Exposure to multiple languages early in development may improve attentional control by requiring children to listen to and process multiple linguistic inputs (Bialystok and Craik, 2022). This enhanced attention control may give bilingual children an advantage on complex tasks that demand high levels of sustained attention (Bialystok, 2024). Despite being related constructs, findings of a bilingual advantage have typically been shown with tasks that require interference suppression as opposed to response inhibition (e.g., Simon and Stroop tasks; Martin-Rhee and Bialystok, 2008; Nayak et al., 2020). However, while some studies support a bilingual advantage in interference suppression, findings remain mixed, with almost twice as many studies documenting an advantage in older children (ages 6–12) than in younger children (Hilchey and Klein, 2011; Planckaert et al., 2023).

These inconsistencies in behavioral findings have led some researchers to suggest that cultural and socioeconomic status (SES) factors such as education, occupation, and income may confound the results (Paap, 2022). A meta-analytic review revealed only a small effect of bilingualism on EF, which disappeared once they adjusted for publication bias (Lowe et al., 2021). However, a Bayesian inference analysis found that bilingual children outperform monolinguals in EF “far more often than chance,” even when controlling for publication bias, year, sample size, and task type (Yurtsever et al., 2023). These conflicting findings highlight the need to investigate bilingualism’s effect using neuroimaging techniques rather than relying solely on behavioral measures (Pliatsikas, 2024). The use of neuroimaging techniques can provide additional information on the underlying cognitive functions that may not be adequately captured by behavioral studies (Morita et al., 2016).

Neuroscience research suggests that as inhibitory control matures, neural activation becomes more specialized and efficient, shifting from broad, global activation to a more localized pattern in the prefrontal cortex (Fiske and Holmboe, 2019; Hwang et al., 2010). This aligns with the neural efficiency hypothesis, which posits that greater proficiency in cognitive tasks becomes more automated by adapting to the demands, thus requiring only specific brain regions and less global activation (Debarnot et al., 2014). This further aligns with the principle of efficient coding which states that the brain works to transmit the maximum amount of information in the way that is most metabolically efficient, leading to reduced neural load for behavioral performance (Zhou et al., 2022). Given this framework, examining brain activity can provide deeper insight into how bilingualism influences inhibitory control at the neural level.

Evidence suggests that bilinguals may develop more efficient attentional control, particularly in tasks requiring interference suppression, where irrelevant information must be ignored. For example, in a response inhibition task, Mehnert et al. (2013) found increased connectivity for children ages 4–6 in the bilateral frontal and parietal cortices during both response and inhibition trials using functional near-infrared neuroimaging (fNIRS). Conversely, the adult participants activated only during the inhibition trials and only within the right frontal and parietal cortex. Their results align with the neural efficiency hypothesis, suggesting that younger children, who have less practice with inhibition, recruit neural networks that are broader and not fully specialized compared with adults who have had more practice and are more proficient. Bilingualism, by providing additional practice in attentional control, may accelerate this neural adaption process, leading to less global activation and greater efficiency during inhibition, even in young children (Li et al., 2023).

Bilingualism has been associated with structural brain changes in both grey and white matter, particularly as second language proficiency increases (García-Pentón et al., 2016; Luk and Pliatsikas, 2016; Pliatsikas, 2020). These structural changes in the brain have been shown to influence functional connectivity patterns (Wang and Tao, 2024) and behavioral outcomes for cognitive skills (Gavett et al., 2018). This may, in turn, contribute to a potential bilingual advantage or more efficient cognitive processes in childhood. Task-based functional connectivity measured with fNIRS assesses how different brain regions interact and coordinate their activity during the performance of a specific task. It reflects the temporal correlation in oxygenated hemoglobin fluctuations between regions, revealing which areas co-activate during task engagement and thus may be functionally linked. This approach helps identify networks of coordinated neural activity that support specific cognitive processes, such as inhibitory control (Linnman et al., 2012; Chong et al., 2025). In the context of inhibitory control, studies examining interference suppression have found increased neural activation in areas of the prefrontal cortex, including the bilateral dorsolateral-prefrontal cortex [dlPFC; Brodmann areas (BAs) 9 and 46], medial prefrontal cortex (mPFC; BA 10) and bilateral inferior frontal gyrus (IFG; BA 45; Peters and Smith, 2020; Tao et al., 2021; Wang and Tao, 2024). These findings highlight the importance of investigating how bilingualism may shape functional connectivity within these networks during interference suppression tasks.

Despite these advances, understanding the functional impact of bilingualism on interference suppression remains challenging, particularly in young children. However, a recent review highlights fNIRS as a promising tool for refining existing theories on the effects of bilingualism and cognition (see Pliatsikas, 2024 for a review). Importantly, the review notes that fNIRS studies have yet to provide consistent evidence on how bilingualism influences brain function, underscoring the need for further research to clarify these effects. For instance, a study found no association between second language on functional brain activation during a task (Moriguchi and Lertladaluck, 2020), whereas other studies have found those positive associations (Arredondo et al., 2022; Xie et al., 2021).

Furthermore, a recent fNIRS study found evidence that suggests that bilingualism may promote neural efficiency in the PFC. It found that bilingual children required fewer cortical resources during a card sort interference/switching task, suggesting that bilinguals require fewer neural resources to achieve similar behavioral performance as monolinguals (Li et al., 2023). Building on these findings, the current study leverages fNIRS to examine functional connectivity within the bilateral dlPFC, mPFC, and bilateral IFG during interference suppression task. This will provide deeper insights into how bilingual and monolingual preschoolers differ in their neural processing of interference suppression.

By incorporating fNIRS alongside behavioral measures of interference suppression in early childhood, the current study aims to enhance our understanding of the bilingual experience of young children during inhibition. Based on conceptualizations of bilingualism promoting attentional control (Bialystok, 2024; Bialystok and Craik, 2022), we hypothesize that bilingual preschoolers will demonstrate similar behavioral performance on the congruent trials requiring less attentional control and better performance on the incongruent trials of a Simon-like interference suppression task than age-matched monolingual preschoolers. We further hypothesize task-state functional connectivity region of interest pattern differences within the prefrontal cortex with bilingual preschoolers, demonstrating more efficient neural processing (i.e., less global connections) than monolingual preschoolers.

## Materials and methods

### Participants

The current study utilized data from 26 preschool aged children recruited from an on-campus childcare from a large public university in the United States of America and the surrounding community. Parents of participating children were asked to complete online questionnaires describing family characteristics and home language use. The current sample included 13 bilingual children (female = 9, mean age = 59.39 months) whose home languages were primarily of East Asian or Ibero-Romantic descent and 13 age matched monolingual English children (female = 5, mean age = 59.22 months). Most participating parents for bilingual children had a bachelor’s degree (*n* = 5) followed by those with a doctorate (*n* = 4) then a master’s degree (*n* = 3). One parent had completed some college. Most participating parents for monolingual children had a bachelor’s degree (*n* = 7) followed by a doctorate (*n* = 2), master’s degree (*n* = 2), and having completed some college (*n* = 2). Second languages spoken at home include Mandarin (*n* = 4), Portuguese (*n* = 3), Spanish (*n* = 3), Korean (*n* = 1), German (*n* = 1), and French (*n* = 1). Descriptive statistics for all continuous and categorical variables are presented in Table 1.

**Table 1:** Demographic characteristics.

Characteristic	Monolingual		Bilingual		Total sample	
	Mean	SD	Mean	SD	Mean	SD
Age (months)	59.22	9.27	59.39	9.27	59.30	9.08
English proficiency (PPVT)	128.7	17.64	115.2	23.23	121.95	21.24
Effortful control	68.25	6.92	65.69	13.19	66.92	10.52
	*n*	%	*n*	%	*n*	%
Sex						
Female	5	38	9	69	14	54
Male	8	62	4	31	12	46
Parent education						
Some college	2	15	1	8	3	12
Bachelors	7	54	5	38	12	46
Masters	2	15	3	23	5	19
Doctorate	2	15	4	31	6	23
Second language						
Mandarin			4	31		
Portuguese			3	23		
Spanish			3	23		
Korean			1	8		
German			1	8		
French			1	8		

Pairwise comparison	Congruent RT		Incongruent RT		*t*(12)	*p*-value
	*M* (ms)	*SD* (ms)	*M* (ms)	*SD* (ms)		
Monolingual	1474.06	285.96	1581.07	370.31	−2.12	0.055
Bilingual	1403.94	323.73	1531.85	413.55	−1.24	0.239

*N* = 26 (*n* = 13 for each group).

Bilingualism can be a complex construct, with definitions varying based on age and study purpose. For example, very young children may be considered bilingual if they receive an appropriate amount of exposure to a second language whereas more weight would be given to the level of fluency in older youth and adults. Due to the wide variety of language pairings present in our sample, we selected our bilingual sample based on exposure to a second language rather than proficiency (Loe and Feldman, 2016; Valicenti-McDermott et al., 2013; Singh et al., 2015). For this study, bilingualism is defined as having at least 25% exposure to a second language and monolingualism is defined as having at least 90% exposure to a first language (Pearson et al., 1993). The present study utilized a language background questionnaire (LBQ) adapted from a phone-based questionnaire (Singh et al., 2015) where parents were asked about the different home languages to which their children were exposed. Bilingual participants in our sample had an average of 74% (45–100%) second language exposure from parents. Monolingual children were marked as zero exposure to a second language. All parents of participants signed an IRB approved consent form and received monetary compensation for participating. Children provided assent and received a book or toy of their choosing.

### Measures

#### Peabody picture vocabulary test—fourth edition

English receptive vocabulary was measured using the Peabody Picture Vocabulary Test—Fourth Edition (PPVT-4) (Dunn and Dunn, 2007) to ensure that all of the children, regardless of their home language, had similar levels of understanding in English. The PPVT-4 is a norm-referenced task that is individually administered by a trained researcher. The task requires the child to choose the picture that best represents the stated word. The child has four picture options to choose from and the task ends when the child misses 8 or more words in a set of 12. The PPVT-4 provides a standardized score based on age and sex.

#### Child’s behavior questionnaire—effortful control

Similarity between monolingual and bilingual parents’ perceptions of various aspects of their children’s behaviors (e.g., inhibitory control, attention, and emotion regulation), was measured using the effortful control subscale of the child’s behavior questionnaire very short form Putnam and Rothbart (2006) and Rothbart et al. (2001). Parents answered 12 questions related to child effortful control (*α* = 0.88; e.g., Is good at following instructions.) and a sum score was computed. This measure was included to examine whether caregivers’ reports of the aforementioned behaviors differed between bilingual and monolingual preschoolers and to provide an additional context beyond our laboratory setting.

#### Spatial conflict arrows (arrows)

**Figure 1:**
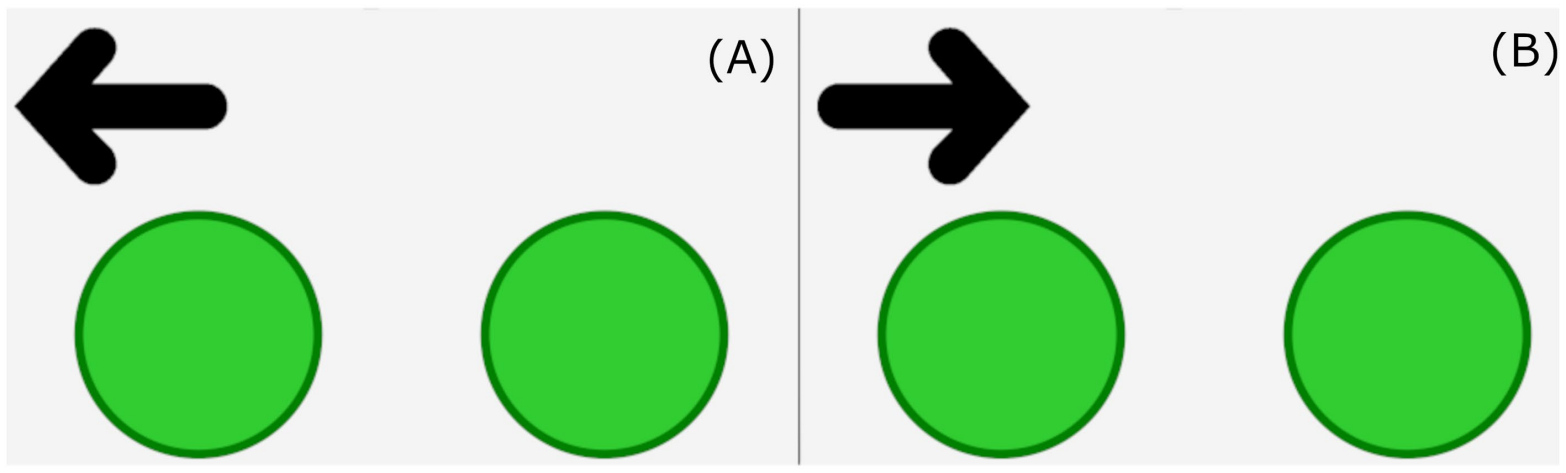
Congruent and incongruent trials for spatial conflict arrows. **(A)** Represents congruent trials and **(B)** represents incongruent trials. Children were required to respond based on the direction the arrow is pointing and not the location of the arrow.

Interference suppression was assessed using the Spatial Conflict Arrows (Arrows; Figure 1) task from the EF Touch battery, a computerized set of tasks developed for children aged 3–5 years (Willoughby et al., 2012a; Willoughby et al., 2012b). This Simon-like task (Simon, 1969) measures interference suppression by requiring children to respond to the direction an arrow is pointing, regardless of its location on the screen. In congruent trials, the arrow appears on the same side it points to (e.g., pointing left on the left side), while in incongruent trials, the arrow appears on the opposite side (e.g., pointing left on the right side). All participants completed the same sequence of 36 trials (19 congruent, 17 incongruent), presented in a single block. The task began with 12 congruent trials, followed by 12 incongruent trials, and ended with a mixed sequence. For the first six participants (split evenly between monolingual and bilingual), trials were presented for 2 s, which was later adjusted to 4 s for the remaining participants to better accommodate younger children.

Accuracy was scored as the proportion of correct responses (0–1.00), and reaction time was recorded in milliseconds. Performance was analyzed separately for congruent and incongruent trials. Group comparisons between bilingual and monolingual children were conducted using Mann–Whitney U tests for accuracy (based on non-normal distributions) and independent-samples *t*-tests for reaction time. Normality was assessed using the Shapiro–Wilk test, appropriate for small sample sizes (*N* < 50) (Ghasemi and Zahediasl, 2012). All statistical analyses were performed using R version 4.4.2 (R Core Team, 2024).

### fNIRS data acquisition and processing

**Figure 2:**
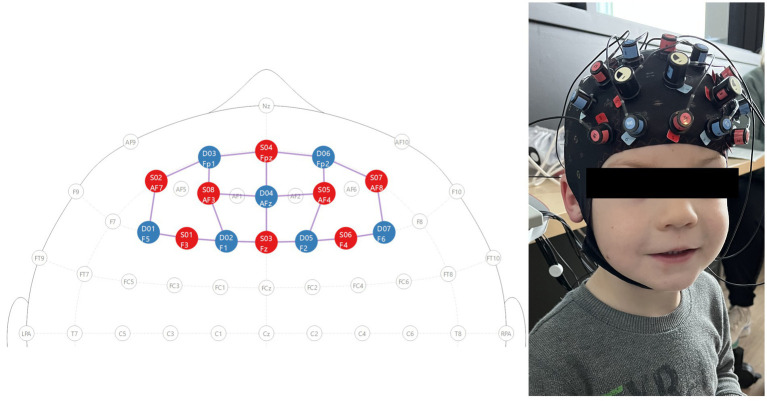
Prefrontal 8 × 8 cap montage. Red circles are source locations and blue circles are detector locations. Location labels follow the EEG 10/10 system. Researchers received verbal consent from the mother and assent from the child to take this photo. Maternal consent was also received to include a deidentified copy of the photo for publication.

fNIRS data were collected in a secured room using a continuous-wave NIRSport2 system (NIRx, Medical Technologies, LLC). A NIRx prefrontal cortex montage containing eight light sources that emit near-infrared light at wavelengths of 760 and 850 nm and seven detectors (Figure 2) was used. This resulted in 20 channels of data being collected. Prior to the neuroimaging session, parents and children provided consent/assent, and efforts were made to ensure participants’ comfort throughout. Prior to data collection, participants were fitted with the cap which was then calibrated to get the best possible signal. If channels had poor connections, the cap was readjusted, and hair was parted to allow for sources and detectors to have more direct contact with the scalp.

Data were exported from the NIRSport2 device into MATLAB version R2022b (The MathWorks Inc., 2022) where the NIRS Brain AnalyzIR Toolbox (Santosa et al., 2018) was used to preprocess the fNIRS data, extract the oxygenated (HbO) and deoxygenated (HbR) hemoglobin values, map the channel coordinates onto the Brodmann areas of interest (BAs 8, 9, 10, 45, and 46) and calculate the inter- and intra-individual correlations between fNIRS channels for both HbO and HbR. The current analyses focused on changes in hemoglobin concentration and functional connectivity for only HbO values as they are more sensitive to task related changes and have a higher signal amplitude than HbR values (Hoshi, 2003; Luke et al., 2021). Prior to preprocessing the fNIRS data, event triggers for all trials were manually input based off an initial task-start trigger from data collection. Trigger timings were calculated following the previously mentioned pattern of trials. These markers were then used for both analyses. First, raw data were visually inspected for noisy channels. Following the visual inspection, raw values were then converted to optical density values and then a temporal derivative distribution repair (Fishburn et al., 2019) was applied to help correct motion artifacts. Next, optical density values were then converted to HbO and HbR values via the modified Beer–Lambert Law (Jacques, 2013). For the functional connectivity analysis, converted data was down sampled from 10 to 1 Hz in order to reduce the impact of serial autocorrelations on analyses (Pinti et al., 2019). However, for the region of interest analysis, data was not down sampled. Finally, due to the unique statistical properties of fNIRS, an autoregressive, iteratively reweighted robust model (pre-whitening) was used to improve model robustness and account for serial dependencies in the data (Barker et al., 2013; Huppert, 2016).

#### Region of interest analysis

Participant neural activity (beta values) were calculated by solving a generalized linear model (GLM) for each channel within every participant for both trial types. Level one of the GLM included individual HbO values during task completion. Level-one analysis also included regressors to model channel activation during each task condition regardless of group. Level-two of the GLM explored main effects of condition for each group. Additionally, between group comparisons for both condition types were examined at this level. The formula for the level-two GLM is displayed here [beta ~ − 1 + group:cond + (1|ID)].

#### Connectivity analysis

Individual level bivariate correlations were calculated between each channel pairing to determine task-state functional connectivity across the prefrontal cortex. The first 24s^1^ for each trial type (i.e., congruent and incongruent) were utilized for the connectivity analyses. Analyzing trial types individually allows us to capture the nuanced activation patterns and gives us a better overall picture of neural efficiency. Prior to the connectivity analyses, channel coordinates were registered to the Colin 27 atlas with a 53 cm circumference. Task-state functional connectivity was assessed using auto-regressive whitened robust correlations between channel pairs, for HbO. Individual-level connectivity patterns were first calculated using the “connectivity” function from the Nirs Brain AnalyzIR toolbox (Santosa et al., 2018). This function calculates an all-to-all connectivity matrix via an autoregressive, robust correlation with a maximum model order of 4x the sampling rate. Group level models were then calculated using the “MixedEffectsConnectivity” function. The formula for the mixed effects model is presented here (R ~ −1 + group:cond) (for additional information on the mathematical procedures and functions see Barker et al., 2013; Lanka et al., 2022; Santosa et al., 2017; Santosa et al., 2018). To determine significance of channel correlations, one-sample t-tests were conducted and a False Discovery Rate (FDR) correction (Benjamini and Hochberg, 1995) was used to account for multiple comparisons.

## Results

### Descriptives

Children in the bilingual and monolingual groups did not differ in age [*t*(26) = −0.04, *p* = 0.97], parent report of effortful control (*U* = 86.50, *z* = 0.46, *p* = 0.663, *r =* 0.09) or receptive English language score as measured by the PPVT-4 [*t*(16.8) = 1.46, *p* = 0.162]. Additionally, bilingual and monolingual groups did not differ in terms of parental education levels [*t*(23.9) = −0.45, *p* = 0.26]. These findings suggests that both bilingual and monolingual children were comparable in age, English receptive language proficiency, parent reported effortful control behavioral scores, and parental education levels, despite differences in language exposure.

### Interference suppression behavioral data

#### Within group differences

To test for a within group interference effect between correct responses for congruent and incongruent trials of the spatial conflict arrows task, paired samples t-tests were conducted for monolingual and bilingual preschoolers. For the bilingual preschoolers, the results were non-significant [*t*(12) = −1.24, *p* = 0.24] suggesting that the bilingual preschoolers did not experience an interference effect during this task. For the monolingual preschoolers, the results were also non-significant [*t*(12) = −2.12, *p* = 0.055], however, as the *p-value* was 0.055 and was approaching significance, this may indicate that there is potentially an interference effect between trial types for monolingual preschoolers and should be tested further with a larger sample.

#### Between group differences

To test if there are group differences in task performance during the spatial conflict arrows task, Mann–Whitney U tests were conducted due to non-normality in the dependent variables based on the results of the Shapiro–Wilk test which is recommended for small sample sizes (Ghasemi and Zahediasl, 2012; Shapiro et al., 1968; *W_Cong_* = 0.832, *p* < 0.001; *W_Inong_* = 0.917, *p* = 0.038; *W_Combined_* = 0.902, *p* = 0.017). Accuracy comparisons between bilingual (*Mdn_Cong_* = 0.89, *IQR* = 0.16; *Mdn_Incong_* = 0.71, *IQR* = 0.18; *Mdn_Combined_* = 0.81, *IQR* = 0.19) and monolingual (*Mdn_Cong_* = 0.84, *IQR* = 0.21; *Mdn_Incong_* = 0.88, *IQR* = 0.29; *Mdn_Combined_* = 0.86, *IQR* = 0.28) preschoolers for task accuracy split by trial type and combined are presented in Table 2. The results indicated that there was not a significant difference in the accuracy between groups (*U_Cong_* = 84*, z* = −0.03*, p* = 0.999*, r* = 0.01; *U_Incong_* = 111.50*, z* = 1.39*, p* = 0.171*, r* = 0.27; *U_Combined_* = 102.50*, z* = 0.93*, p* = 0.368*, r* = 0.18) for congruent and incongruent tasks. To test if there are group differences between bilingual (*M_Cong_* = 1403.94 ms, *SD* = 323.73 ms; *M_Incong_* = 1531.85 ms, *SD* = 413.55 ms; *M_Combined_* = 1415.27 ms, *SD* = 343.51 ms) and monolingual (*M_Cong_* = 1474.06 ms, *SD* = 285.96 ms; *M_Incong_* = 1581.07 ms, *SD* = 370.31 ms; *M_Combined_* = 1484.10 ms, *SD* = 374.36 ms) preschoolers in task reaction time Welch’s two-sample t-tests were conducted as reaction time was normally distributed (*W_Cong_* = 0.944, *p* = 0.169; *W_Incong_* = 0.981, *p* = 0.888; *W_Combined_* = 0.926, *p* = 0.061). Reaction time comparisons between groups are presented in Table 3. The results for the t-test indicated that there was also not a significant difference between groups [*t_Cong_*(23.6) = 0.59, *p* = 0.564, *d* = 0.23; *t_Incong_*(23.7) = 0.32, *p* = 0.752, *d* = 0.13; *t_Combined_*(23.8) = 0.49, *p* = 0.630, *d* = 0.19] for the congruent and incongruent tasks. This Suggests that both groups were able to respond similarly on both congruent and incongruent tasks of the Simon-like task measuring interference suppression.

**Table 2:** Group comparisons of response accuracy for congruent and incongruent trials.

Trial type	Monolingual (*n* = 13)		Bilingual (*n* = 13)		*U*(24)	Z-score	*p*-value
	Mdn	IQR	Mdn	IQR			
Congruent accuracy	0.84	0.21	0.89	0.16	84	−0.03	>0.999
Incongruent accuracy	0.88	0.29	0.71	0.18	111.50	1.39	0.171
Combined accuracy	0.86	0.22	0.81	0.19	102.50	0.93	0.368

Groups were compared via Mann–Whitney U tests.

**Table 3:** Group comparisons of reaction time for correct congruent and incongruent trials.

Trial type	Monolingual (*n* = 13)		Bilingual (*n* = 13)		*t*(23.6)	Cohen’s d	*p*-value
	M	SD	M	SD			
Congruent RT	1474.06	285.96	1403.94	323.73	0.59	0.23	0.564
Incongruent RT	1581.07	370.31	1531.85	413.55	0.32	0.13	0.752
Combined RT	1484.10	374.36	1415.27	343.51	0.49	0.19	0.630

Groups were compared via Welch’s Two Sample *t*-test. Reaction times are in milliseconds.

### Region of interest analysis results

To evaluate how bilingual and monolingual preschoolers differ in patterns of neural recruitment during interference suppression, a generalized linear model analysis was conducted. For congruent trials, neither monolingual or bilingual participants had significant activation for any channels at an FDR corrected *q-value* of 0.05. For incongruent trials, only the monolingual participants had significant activation in channels S1-D2, *β* = 12.49, *SE* = 3.46, *t*(46) = 3.61, *q = 0*.04, and S4-D5, *β* = 16.25, *SE* = 3.39, *t*(46) = 4.79, *q < 0*.01.

For congruent trials, there were no significant differences in channel activation at a *q* < 0.05 between monolingual and bilingual preschoolers. Channels S1-D2*, β* = 12.49, *SE* = 3.46, *t*(46) = 3.61, *q = 0*.01, and S4-D5*, β* = 16.25, *SE* = 3.39, *t*(46) = 4.79, *q < 0*.001.

**Figure 3:**
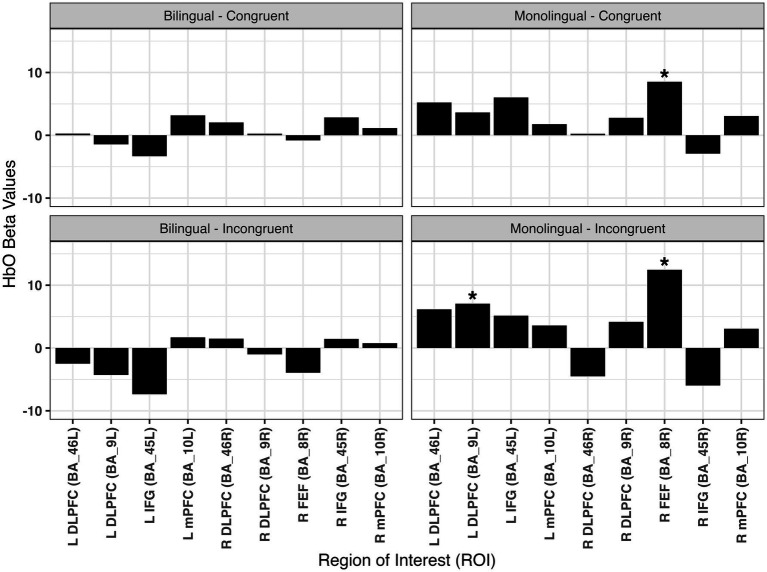
Beta values by region of interest. *q < 0.05. Channel weights for each region of interest are located in Supplementary Table 1; L DLPFC, Left Dorsolateral Prefrontal Cortex; L IFG, Left Inferior Frontal Gyrus; L mPFC, Left Medial Prefrontal Cortex; R DLPFC, Right Dorsolateral Prefrontal Cortex; R FEF, Right Frontal Eye Fields; R IFG, Right Inferior Frontal Gyrus; R mPFC, Right Medial Prefrontal Cortex.

**Figure 4:**
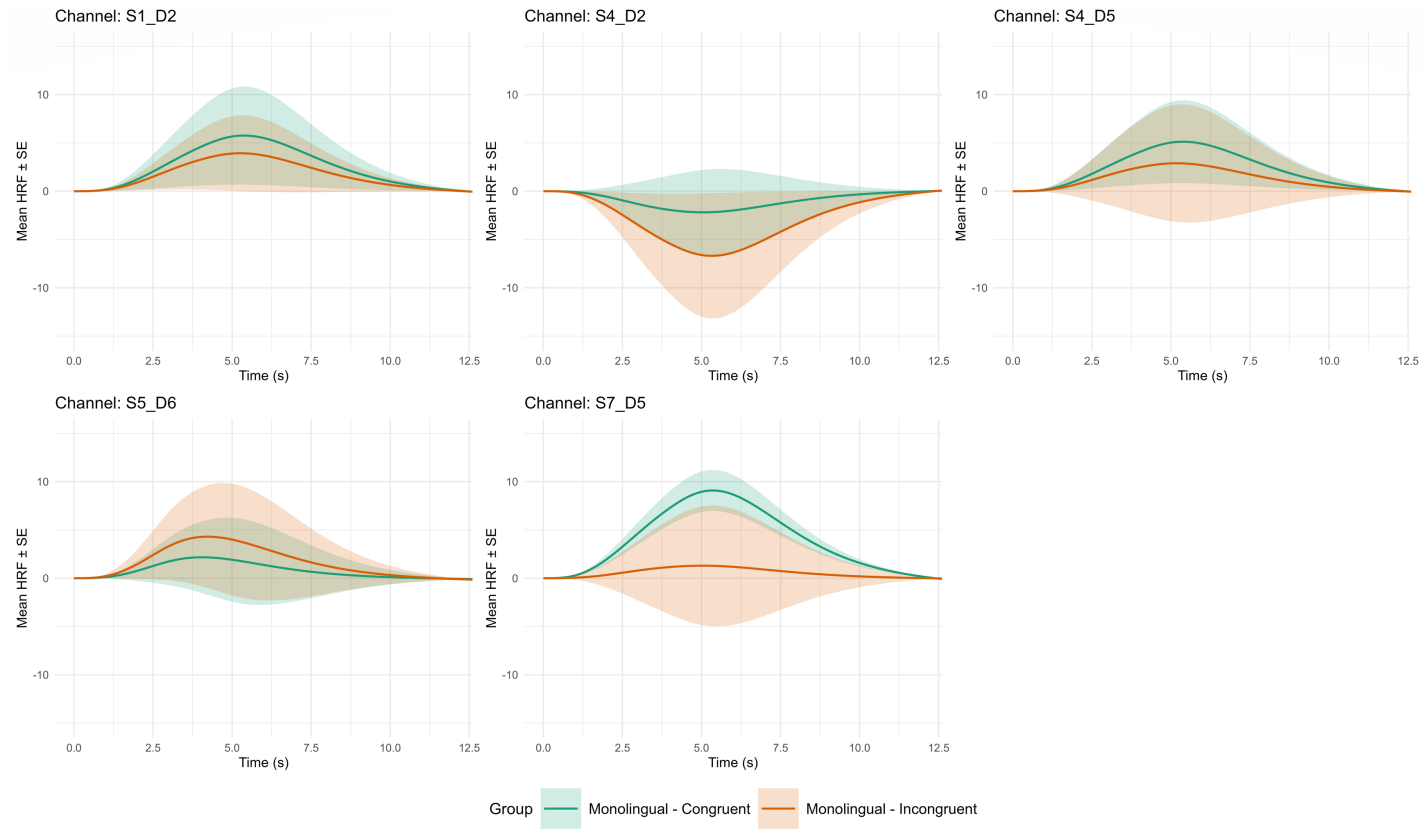
Average hemodynamic response function by channel for significant group × congruent pairings. Error bars are represented by ± 1 SE; Channel S1_D2 covers part of the Left Dorsolateral Prefrontal Cortex (BA_9L); Channel S4_D2 covers part of the Right Frontal Eye Fields (BA_8R); Channel S5_D6 covers part of the Right Medial Prefrontal Cortex (BA_10R); Channel S7_D5 covers part of the Right Dorsolateral Prefrontal Cortex (BA_9R).

**Figure 5:**
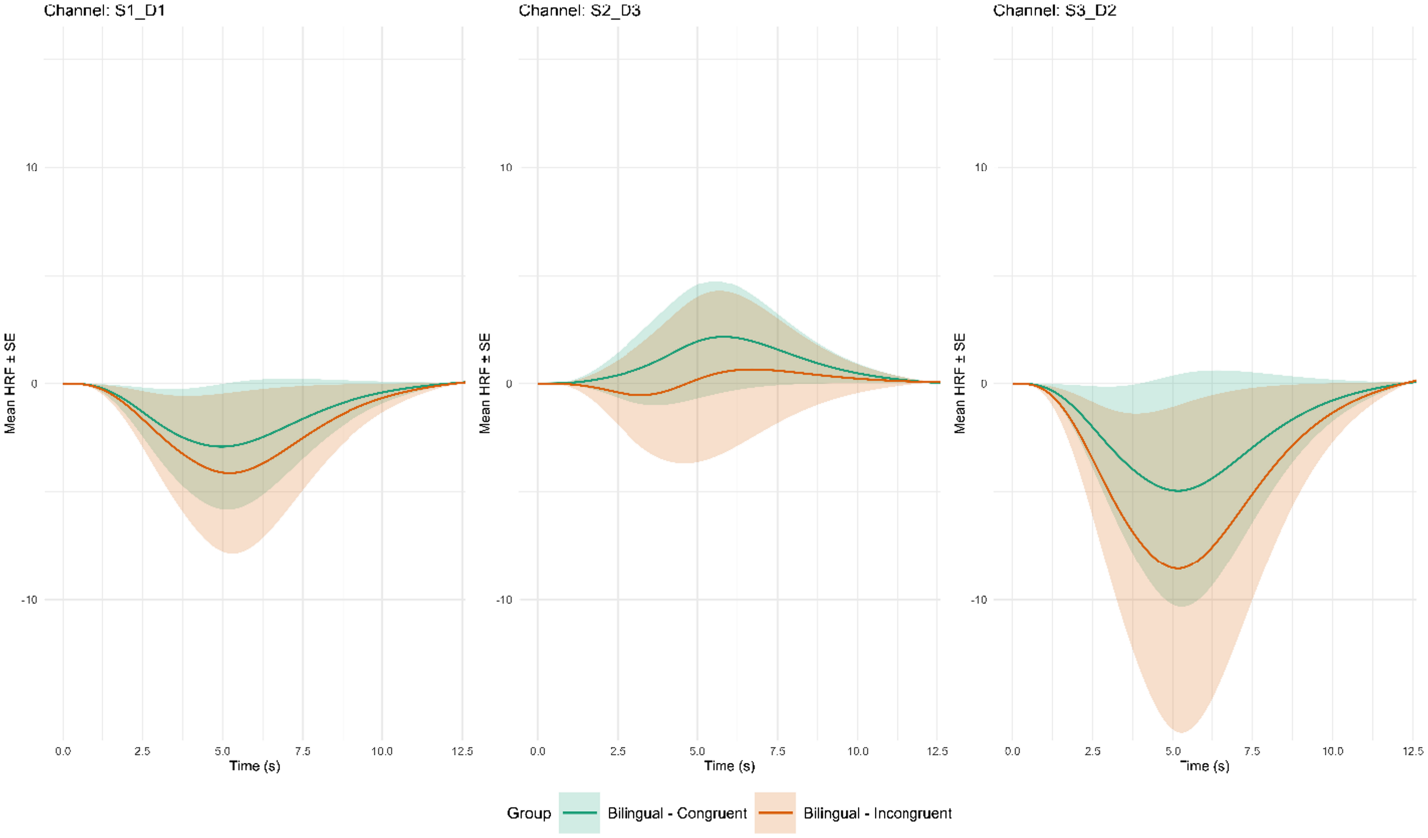
Average hemodynamic response function by channel for significant group × incongruent pairings. Error bars are represented by ± 1 SE; Channel S1_D1 covers part of the Left Inferior Frontal Gyrus (BA_45L); Channel S2_D3 covers part of the Left Medial Prefrontal Cortex (BA_10L); Channel S3_D2 covers part of the Left Dorsolateral Prefrontal Cortex (BA_9L).

A bar plot for fNIRS channels averaged into regions of interest based on the channel weights (Supplementary Table 1) is displayed in Figure 3. Each bar plot represents a different group (Bilingual/Monolingual) by trial type (Congruent/Incongruent) pairing. The canonical hemodynamic response function for significant (*p* < 0.05) group by trial type for each channel is displayed in Figure 4 for monolingual preschoolers and Figure 5 for bilingual preschoolers. Supplementary results at an uncorrected *p* < 0.05 are also included in Supplementary information. These results did not meet the significance threshold after correcting for multiple comparisons. However, they may display important trends which are valuable for future research with larger sample sizes. We are cautious to overinterpret these uncorrected results but hope that sharing these patterns will guide future investigations in this area.

### Functional connectivity results

**Figure 6:**
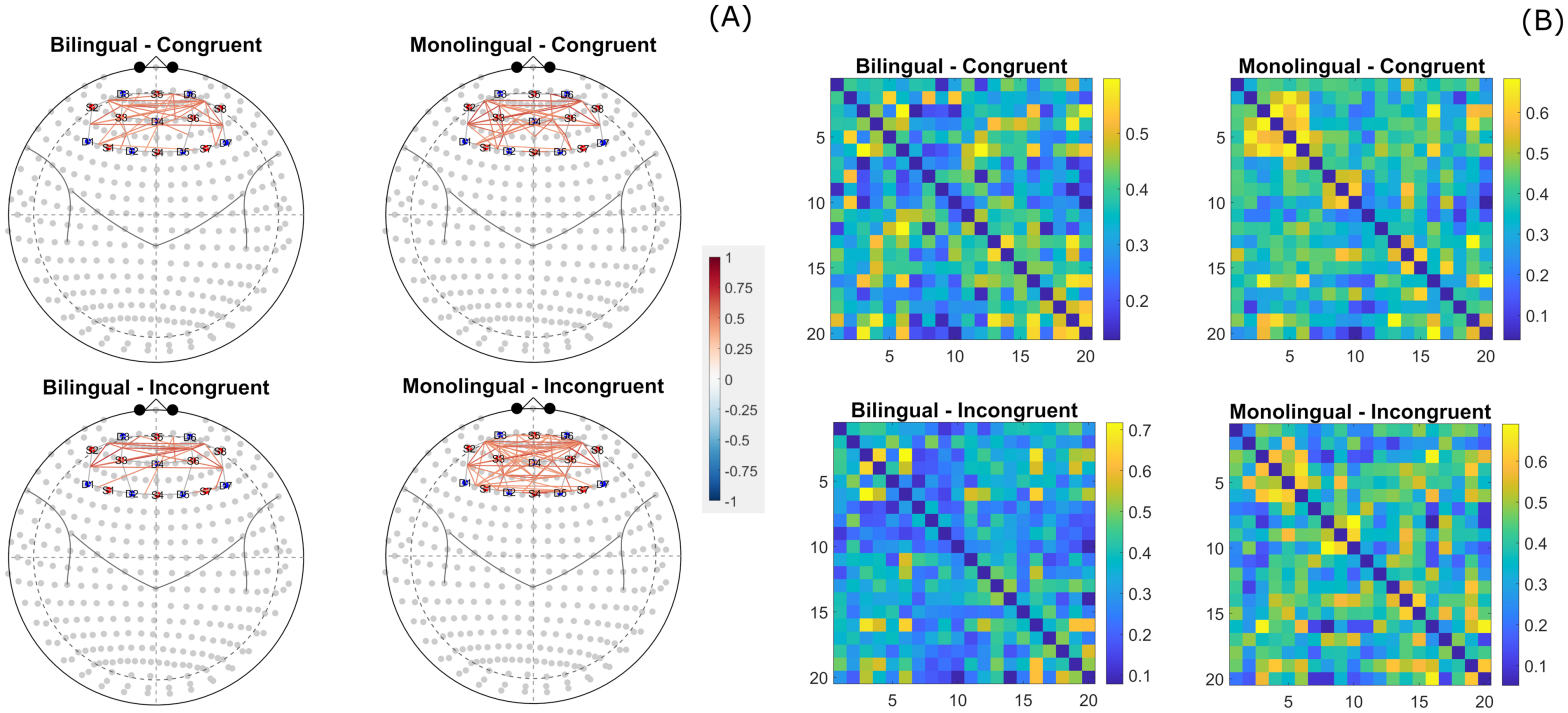
Task-state functional connectivity patterns for oxygenated hemoglobin. **(A)** Displays task-state functional connectivity patterns for all preschoolers split by group and trial type. **(B)** Displays task-state functional connectivity correlation matrices split by group and trial type. Correlation patterns are displayed on a Mercator projected 2D atlas for each group x trial type pairing.

To test our hypothesis that bilingual children would show more efficient neural processing during interference suppression, we examined group differences in task-related functional connectivity within the prefrontal cortex. Specifically, we expected bilingual preschoolers to exhibit more localized connectivity patterns reflecting efficient recruitment of executive control regions, while monolingual preschoolers were hypothesized to show broader, less focused connectivity, indicative of greater or less efficient resource recruitment. Bilingual preschoolers exhibited more localized connectivity with significant correlations primarily observed in the left and right hemispheres of the dorsolateral (BAs 9 and 46) and medial (BA10) PFC. At an FDR corrected *q-value* of 0.001, bilingual children showed 28 significant correlations during congruent trials and 21 significant correlations for incongruent trials. However, there were relatively few connections between the dlPFC/mPFC to the Frontal Eye-fields (FEF; BA 8) and the left/right IFG (BA 45) suggesting more efficient recruitment of core executive function regions during task performance. Conversely, monolingual preschoolers exhibited a more broadly distributed pattern of functional connectivity. Monolinguals showed 35 significant connections during congruent trials and 63 significant correlations for incongruent trials, across the PFC, including stronger connections to the FEF and IFG, suggesting less efficient and more broadly distributed engagement of PFC, potentially requiring the co-activation of multiple regions to support task demands during interference suppression (see Figure 6).

Together these results suggest bilingual and monolingual preschoolers engage distinct functional connectivity patterns during task-state interference suppression Bilingual preschoolers exhibited a more localized connectivity pattern particularly between regions of the left and right dlPFC and mPFC which may reflect a more efficient recruitment of neural resources. In contrast, monolingual showed broader PFC connectivity, particularly with attention control regions FEF and cognitive control IFG, suggesting greater network-wide engagement during the task. To see a full table of channel weights for Brodmann Areas and channel pairing correlation values (see Supplemental material).

## Discussion

The current study aimed to enhance our understanding of the bilingual experience of young children in relation to executive function by examining both behavioral performance and neural processing during a Simon-like interference suppression task using fNIRS. We focused on the interference suppression aspect of interference suppression following conceptualizations of bilingualism promoting attentional control (Bialystok, 2024; Bialystok and Craik, 2022). Our hypotheses were two-fold. First, for the behavioral data, we hypothesized that monolingual and bilingual preschoolers would perform similarly on the congruent interference suppression task which did not require suppression of information, and that bilingual preschoolers would perform better than monolingual preschoolers on the incongruent task that required more attentional control. Second, for the neuroimaging data, we hypothesized that compared to monolingual preschoolers, bilingual preschoolers would demonstrate more efficient neural processing resulting in less global connections during interference suppression.

As hypothesized, bilingual and monolingual preschoolers performed similarly on congruent trials. Conversely, our lack of significant differences for accuracy and response time on incongruent trials are inconsistent with our hypothesis that bilingual children would outperform the monolingual children on incongruent trials. However, these findings are consistent with recent literature that reported no bilingual group advantage in behavioral performance during either the congruent or incongruent trials in an age-appropriate interference suppression task, indicated by the similarity in performance on accuracy and response time (Lowe et al., 2021). Similarities in behavioral data findings may support the perspective that children need to be older with more refined interference suppression skills having been developed before behavioral performance differences are found between monolinguals and bilinguals. Indeed, a systematic review conducted found that for interference suppression, only 38% of studies found a bilingual advantage when using a Simon-like task during the critical period (i.e., younger than 6 years old) compared to 60% who found an advantage in the older, post-critical period age group (i.e., 6–12 years old; Planckaert et al., 2023). Following the theory that attention control is the underlying mechanism that could contribute to a bilingual advantage, the tasks, while age-appropriate, may not have been complex enough to require high levels of attention (Bialystok, 2024). While these explanations should be examined further, it may be that there is not a detectable inhibition behavioral difference between monolingual and bilingual preschoolers. Indeed, a recent meta-analysis suggests that research studies that report a bilingual advantage are based on small effect sizes with the findings moderated by SES and the quality of the research design (Lowe et al., 2021). They suggest addressing these limitations through large sample sizes, improved methodological rigor and moving beyond measuring bilingualism/monolingualism as a dichotomous variable (Lowe et al., 2021). As bilingualism is not a static, monolithic trait, bilingual children vary in their age of language acquisition, proficiency in each language, switching between languages and the context for language interactions (De Bruin, 2019). More detailed and nuanced information about bilingual participants and their language experiences could improve our understanding of the effect of bilingualism on the complex construct of executive functioning.

### Region of interest and functional connectivity

Interestingly, despite the lack of differences in behavioral performance, we found strong evidence for differences in ROI activation and task-state functional connectivity patterns. Regarding ROI activation, in comparison with bilinguals, there was the greatest increase in activation for monolinguals during incongruent trials in channel S1-D2 (FEF; BA8) and S4-D5 (BA9-dorsomedial PFC and BA10-frontopolar PFC). FEF have been implicated in eye/gaze control and decision-making driven by uncertainty (Dadario et al., 2023). The dorsomedial PFC has shown involvement with response-, decision-, and strategic-control (Venkatraman et al., 2009) and the frontopolar PFC has been associated with metacognition during decision making (Qiu et al., 2018) and also in processing concurrent potential behavioral plans during decision making (Koechlin and Hyafil, 2007).

Also, while exhibiting more localized prefrontal connectivity patterns, bilingual preschoolers showed similar behavioral performance to monolingual preschoolers in both task accuracy and reaction time. This, in conjunction with the aforementioned reduced activation in key decision-making areas of the PFC, suggests that bilingual children may engage a more efficient prefrontal network during interference suppression rather than relying on widespread cortical recruitment. Bilingual children may have more practice with and proficiency in attentional control due to their experience with processing multiple languages (Bialystok and Craik, 2022), leading to adapted neural activation patterns aligning with the neural efficiency hypothesis (Debarnot et al., 2014).

Our findings are supported by Li et al. (2023) who found that bilingual preschoolers showed similar behavioral performance to monolingual preschoolers in a switching/interference suppression task, however, the bilingual preschoolers activated only 11 channels compared to the 15 of the monolingual children suggesting more efficient executive function processing for the bilingual children. Additionally, similar to our results, monolingual children significantly activated the FEF region whereas bilingual children did not (Li et al., 2023). The FEF region is heavily involved in the allocation of attention to relevant stimuli (Corbetta and Shulman, 2002). The reduced connectivity to the FEF in bilingual children may reflect greater efficiency within attentional control, requiring less engagement of this region to allocate attention to relevant stimuli. This finding provides some support for the idea that bilingualism facilitates neural efficiency in executive function. Conversely, monolingual children, who have less experience managing competing linguistic inputs, may require broader and less specialized patterns when suppressing conflicting stimuli.

Our results suggest that bilingual and monolingual preschoolers recruit different neural networks when suppressing irrelevant information. The bilingual children exhibited lower activation across prefrontal regions, as reflected in smaller beta values from the GLM analysis for both congruent and incongruent trials, along with more localized task-based connectivity patterns. Despite these differences, they achieved comparable accuracy and reaction times to monolingual children. Together, these findings suggest that bilingual children may engage a more efficient and targeted prefrontal network during interference suppression (incongruent trials), requiring fewer cortical resources to meet the same cognitive demands. These reduced activation levels and specialized connectivity patterns may reflect a more refined neural strategy rather than broad, distributed prefrontal recruitment. Over time, this efficiency in executive function may contribute to cognitive advantages, particularly in academic settings where attentional control and cognitive flexibility are essential for learning. As bilingual children continue to develop, their ability to manage competing linguistic inputs, especially for those who are highly proficient in their second language, may enhance their capacity to process new, complex information with reduced reliance on broader cognitive resources (Hansen et al., 2025).

## Limitations and future directions

The current study should be interpreted in light of several limitations. First, the sample size was small (13 per group) which limits statistical power to detect smaller effect sizes especially for group differences in behavioral results. However, our sample size was comparable to multiple others looking at bilingual and monolingual preschoolers (Arredondo et al., 2017; Li et al., 2023; Moriguchi and Lertladaluck, 2020; Okanda et al., 2010). Future studies should utilize larger group sizes to increase power. Second, the sample was recruited from a single large public university and the surrounding community which may limit the generalizability of the results to other regions. The language experiences for both bilingual and monolingual preschoolers are likely to be different based on region (i.e., rural/urban; McCoy et al., 2016; Yang et al., 2025) and should be examined further. Sampling from multiple diverse locations to increase generalizability would help address this limitation. Third, short separation channels (SSCs) were not utilized in the current study which introduces the risk of superficial noise contaminating the fNIRS signal. Future studies should consider the use of SSCs, however, it should be noted that for young children (i.e., 3 years old) 1 cm SSCs may include some cortical contribution (Pinti et al., 2024) increasing the risk of removing true cortical activity when analyzing the data which warrants caution when using SSCs with young children. Fourth, the bilingual group included a wide variety of home languages which may bias results as language pairings may impact functional brain activation differently during interference suppression due to concepts such as language distance (Leivada et al., 2024). Future studies may benefit from limiting the range of language pairings or systematically accounting for language distance to better isolate bilingualism-related effects. Fifth, we were unable to fully control for child sex due to the smaller sample size. While our matching procedure prioritized age, this resulted in unequal sex distributions across groups. Balancing sex across conditions in future work would allow for better control of this variable. Finally, the trial blocks for the connectivity analysis were only 24 s per condition. Although this approach ensured consistency across participants, longer task blocks may improve the reliability of connectivity estimates (Wang et al., 2017).

## Conclusion

The current study used fNIRS neuroimaging and behavioral data to examine inhibitory control in bilingual and monolingual preschool-aged children. Region of interest (ROI) GLM and task state functional connectivity analyses across the prefrontal cortex were analyzed for an interference suppression Simon-like task for 13 bilingual and 13 monolingual children. While bilingual and monolingual child performed similarly in terms of task accuracy and reaction time, ROI and functional connectivity analyses revealed that bilingual preschoolers exhibited a more localized prefrontal connectivity pattern compared to monolinguals, which may indicate greater neural efficiency during interference suppression processing compared to their monolingual peers. Greater efficiency in interference suppression may reflect adaptations in attention control due to experience with bilingualism, potentially supporting broader cognitive development. Further research is needed to explore how these neural differences evolve over time and whether they translate into long-term cognitive or academic advantages.

## Data Availability

The raw data supporting the conclusions of this article will be made available by the authors, without undue reservation.

## Footnotes

1 To ensure consistency in our time-based connectivity analysis, we constrained the analysis window to an equal duration (24 s) across all participants, regardless of trial timing. Although the first six participants completed 12 trials at 2 s each, and the remaining participants completed 12 trials at 4 s each, the same 24-s time window was used for all. A sensitivity analysis excluding the first 6 participants revealed a global decrease of connections for all trial type x group combinations while maintaining similar patterns between monolingual and bilingual children. This global decrease likely represents a loss of statistical power, thus, we retained the full sample size of 26 participants for the final analyses.
