# Supplementary material for: Examining the role of early bilingualism on interference suppression and prefrontal connectivity
Source: Front Integr Neurosci. 2025 Dec 17;19:1591250. doi: 10.3389/fnint.2025.1591250 (PMC12753874; doi:10.3389/fnint.2025.1591250)
Supplement: Supplementary file 1 [file Table_1.docx]

**SI Table 1**

*Channel coverage for Brodmann Areas*

| Weight | Covered Region | Channel Pairing |
| --- | --- | --- |
| 0.571538 | BA-45_L | S1_D1 |
| 0.232652 | BA-9_L | S1_D2 |
| 0.614303 | BA-46_L | S2_D1 |
| 0.283793 | BA-10_L | S2_D3 |
| 0.070763 | BA-9_L | S3_D2 |
| 0.200422 | BA-10_L | S3_D3 |
| 0.419189 | BA-9_L | S3_D4 |
| 0.055646 | BA-9_L | S4_D2 |
| 0.115784 | BA-8_R | S4_D4 |
| 0.6381 | BA-8_R | S4_D5 |
| 0.338761 | BA-10_L | S5_D3 |
| 0.04816 | BA-10_R | S5_D4 |
| 0.398733 | BA-10_R | S5_D6 |
| 0.425411 | BA-9_R | S6_D4 |
| 0.073014 | BA-9_R | S6_D5 |
| 0.200261 | BA-10_R | S6_D6 |
| 0.277521 | BA-9_R | S7_D5 |
| 0.545884 | BA-45_R | S7_D7 |
| 0.332173 | BA-46_R | S8_D6 |
| 0.463943 | BA-46_R | S8_D7 |
| *Note.* BA = Brodmann Area; L = Left Hemisphere; R = Right hemisphere; S = Source; D = Detector | | |
