# Supplementary material for: Examining the role of early bilingualism on interference suppression and prefrontal connectivity
Source: Front Integr Neurosci. 2025 Dec 17;19:1591250. doi: 10.3389/fnint.2025.1591250 (PMC12753874; doi:10.3389/fnint.2025.1591250)
Supplement: Supplementary file 2 [file Table_2.docx]

**SI Table 2**

*Significant Correlations for Bilingual Preschoolers – Congruent Trials*

| Group | *R* | *Z* | *t* | *p-value* | *q-value* | Channel Pairings | ROI Pairings |
| --- | --- | --- | --- | --- | --- | --- | --- |
| Bilingual | 0.431 | 0.461 | 4.372 | 6.59E-05 | 9.89E-04 | S1_D1 - S5_D3 | BA-45_L - BA-10_L |
| Bilingual | 0.439 | 0.471 | 4.583 | 3.28E-05 | 6.54E-04 | S1_D1 - S6_D4 | BA-45_L - BA-9_R |
| Bilingual | 0.395 | 0.417 | 4.397 | 6.07E-05 | 9.55E-04 | S1_D1 - S7_D5 | BA-45_L - BA-9_R |
| Bilingual | 0.498 | 0.546 | 5.369 | 2.27E-06 | 1.11E-04 | S1_D1 - S8_D6 | BA-45_L - BA-46_R |
| Bilingual | 0.551 | 0.620 | 4.487 | 4.51E-05 | 7.81E-04 | S1_D2 - S3_D2 | BA-9_L - BA-9_L |
| Bilingual | 0.598 | 0.690 | 5.477 | 1.56E-06 | 1.11E-04 | S2_D1 - S3_D3 | BA-46_L - BA-10_L |
| Bilingual | 0.527 | 0.586 | 5.389 | 2.12E-06 | 1.11E-04 | S2_D1 - S6_D6 | BA-46_L - BA-10_R |
| Bilingual | 0.441 | 0.474 | 4.547 | 3.70E-05 | 6.72E-04 | S2_D1 - S8_D6 | BA-46_L - BA-46_R |
| Bilingual | 0.583 | 0.668 | 6.667 | 2.38E-08 | 4.64E-06 | S2_D1 - S8_D7 | BA-46_L - BA-46_R |
| Bilingual | 0.457 | 0.493 | 4.856 | 1.32E-05 | 4.28E-04 | S2_D3 - S3_D2 | BA-10_L - BA-9_L |
| Bilingual | 0.486 | 0.531 | 4.452 | 5.07E-05 | 8.41E-04 | S2_D3 - S3_D3 | BA-10_L - BA-10_L |
| Bilingual | 0.523 | 0.581 | 4.578 | 3.34E-05 | 6.54E-04 | S2_D3 - S5_D6 | BA-10_L - BA-10_R |
| Bilingual | 0.497 | 0.545 | 4.666 | 2.49E-05 | 5.87E-04 | S2_D3 - S6_D5 | BA-10_L - BA-9_R |
| Bilingual | 0.575 | 0.655 | 5.894 | 3.64E-07 | 4.73E-05 | S2_D3 - S8_D6 | BA-10_L - BA-46_R |
| Bilingual | 0.399 | 0.423 | 4.562 | 3.52E-05 | 6.54E-04 | S3_D2 - S5_D6 | BA-9_L - BA-10_R |
| Bilingual | 0.594 | 0.684 | 5.012 | 7.73E-06 | 2.74E-04 | S3_D3 - S5_D4 | BA-10_L - BA-10_R |
| Bilingual | 0.545 | 0.611 | 4.715 | 2.11E-05 | 5.49E-04 | S3_D3 - S6_D6 | BA-10_L - BA-10_R |
| Bilingual | 0.472 | 0.513 | 4.394 | 6.12E-05 | 9.55E-04 | S3_D3 - S8_D6 | BA-10_L - BA-46_R |
| Bilingual | 0.532 | 0.593 | 5.367 | 2.28E-06 | 1.11E-04 | S3_D3 - S8_D7 | BA-10_L - BA-46_R |
| Bilingual | 0.430 | 0.460 | 4.572 | 3.41E-05 | 6.54E-04 | S4_D2 - S5_D6 | BA-9_L - BA-10_R |
| Bilingual | 0.556 | 0.628 | 5.732 | 6.41E-07 | 7.14E-05 | S5_D6 - S6_D4 | BA-10_R - BA-9_R |
| Bilingual | 0.573 | 0.652 | 4.578 | 3.33E-05 | 6.54E-04 | S5_D6 - S8_D6 | BA-10_R - BA-46_R |
| Bilingual | 0.495 | 0.543 | 4.463 | 4.88E-05 | 8.27E-04 | S6_D4 - S8_D6 | BA-9_R - BA-46_R |
| Bilingual | 0.568 | 0.645 | 4.572 | 3.40E-05 | 6.54E-04 | S6_D6 - S8_D6 | BA-10_R - BA-46_R |
| Bilingual | 0.553 | 0.623 | 4.774 | 1.73E-05 | 5.20E-04 | S6_D6 - S8_D7 | BA-10_R - BA-46_R |
| Bilingual | 0.488 | 0.534 | 4.728 | 2.02E-05 | 5.43E-04 | S7_D7 - S8_D6 | BA-45_R - BA-46_R |
| Bilingual | 0.533 | 0.595 | 4.395 | 6.10E-05 | 9.55E-04 | S7_D7 - S8_D7 | BA-45_R - BA-46_R |
| Bilingual | 0.558 | 0.629 | 5.375 | 2.23E-06 | 1.11E-04 | S8_D6 - S8_D7 | BA-46_R - BA-46_R |
| *Note.* BA = Brodmann Area; S = Source; D = Detector; L = Left Hemisphere; R = Right Hemisphere; All correlations are significant at an FDR corrected q-value of .001. | | | | | | | |

**SI Table 3**

*Significant Correlations for Bilingual Preschoolers – Incongruent Trials*

| Group | *R* | *Z* | *t* | *p-value* | *q-value* | Channel Pairings | ROI Pairings |
| --- | --- | --- | --- | --- | --- | --- | --- |
| Bilingual | 0.490 | 0.537 | 5.275 | 3.14E-06 | 1.65E-04 | S1_D1 - S8_D6 | BA-45_L - BA-46_R |
| Bilingual | 0.657 | 0.788 | 6.581 | 3.23E-08 | 5.04E-06 | S2_D1 - S2_D3 | BA-46_L - BA-10_L |
| Bilingual | 0.717 | 0.902 | 7.156 | 4.24E-09 | 1.10E-06 | S2_D1 - S3_D3 | BA-46_L - BA-10_L |
| Bilingual | 0.680 | 0.828 | 7.624 | 8.17E-10 | 6.38E-07 | S2_D1 - S6_D6 | BA-46_L - BA-10_R |
| Bilingual | 0.532 | 0.593 | 5.698 | 7.23E-07 | 5.64E-05 | S2_D1 - S8_D6 | BA-46_L - BA-46_R |
| Bilingual | 0.543 | 0.609 | 6.083 | 1.87E-07 | 2.08E-05 | S2_D1 - S8_D7 | BA-46_L - BA-46_R |
| Bilingual | 0.557 | 0.629 | 5.272 | 3.18E-06 | 1.65E-04 | S2_D3 - S3_D3 | BA-10_L - BA-10_L |
| Bilingual | 0.571 | 0.650 | 4.979 | 8.67E-06 | 3.56E-04 | S2_D3 - S6_D6 | BA-10_L - BA-10_R |
| Bilingual | 0.570 | 0.647 | 5.829 | 4.57E-07 | 3.96E-05 | S2_D3 - S8_D6 | BA-10_L - BA-46_R |
| Bilingual | 0.572 | 0.650 | 5.010 | 7.80E-06 | 3.38E-04 | S3_D3 - S5_D3 | BA-10_L - BA-10_L |
| Bilingual | 0.647 | 0.771 | 5.947 | 3.02E-07 | 2.94E-05 | S3_D3 - S6_D6 | BA-10_L - BA-10_R |
| Bilingual | 0.541 | 0.605 | 5.478 | 1.55E-06 | 9.32E-05 | S3_D3 - S8_D7 | BA-10_L - BA-46_R |
| Bilingual | 0.428 | 0.457 | 4.543 | 3.74E-05 | 9.42E-04 | S4_D2 - S5_D6 | BA-9_L - BA-10_R |
| Bilingual | 0.437 | 0.469 | 4.524 | 4.00E-05 | 9.43E-04 | S5_D3 - S6_D6 | BA-10_L - BA-10_R |
| Bilingual | 0.551 | 0.620 | 4.545 | 3.72E-05 | 9.42E-04 | S5_D4 - S6_D6 | BA-10_R - BA-10_R |
| Bilingual | 0.517 | 0.573 | 5.229 | 3.67E-06 | 1.79E-04 | S5_D6 - S6_D4 | BA-10_R - BA-9_R |
| Bilingual | 0.624 | 0.731 | 5.184 | 4.29E-06 | 1.97E-04 | S6_D6 - S8_D6 | BA-10_R - BA-46_R |
| Bilingual | 0.618 | 0.722 | 5.528 | 1.31E-06 | 8.93E-05 | S6_D6 - S8_D7 | BA-10_R - BA-46_R |
| Bilingual | 0.507 | 0.559 | 4.609 | 3.01E-05 | 8.39E-04 | S7_D5 - S7_D7 | BA-9_R - BA-45_R |
| Bilingual | 0.577 | 0.658 | 4.860 | 1.30E-05 | 4.33E-04 | S7_D7 - S8_D7 | BA-45_R - BA-46_R |
| Bilingual | 0.514 | 0.568 | 4.852 | 1.33E-05 | 4.33E-04 | S8_D6 - S8_D7 | BA-46_R - BA-46_R |
| *Note.* BA = Brodmann Area; S = Source; D = Detector; L = Left Hemisphere; R = Right Hemisphere; All correlations are significant at an FDR corrected q-value of .001. | | | | | | | |
