# Supplementary material for: Examining the role of early bilingualism on interference suppression and prefrontal connectivity
Source: Front Integr Neurosci. 2025 Dec 17;19:1591250. doi: 10.3389/fnint.2025.1591250 (PMC12753874; doi:10.3389/fnint.2025.1591250)
Supplement: Supplementary file 3 [file Table_3.docx]

**SI Table 4**

*Significant Correlations for Monolingual Preschoolers – Congruent Trials*

| Group | *R* | *Z* | *t* | *p-value* | *q-value* | Channel Pairings | ROI Pairings |
| --- | --- | --- | --- | --- | --- | --- | --- |
| Monolingual | 0.422 | 0.450 | 4.368 | 6.68E-05 | 9.64E-04 | S1_D1 - S5_D4 | BA-45_L - BA-10_R |
| Monolingual | 0.529 | 0.589 | 5.730 | 6.45E-07 | 2.52E-05 | S1_D1 - S6_D4 | BA-45_L - BA-9_R |
| Monolingual | 0.421 | 0.449 | 4.732 | 2.00E-05 | 3.80E-04 | S1_D1 - S7_D5 | BA-45_L - BA-9_R |
| Monolingual | 0.472 | 0.512 | 5.035 | 7.15E-06 | 1.74E-04 | S1_D1 - S8_D6 | BA-45_L - BA-46_R |
| Monolingual | 0.454 | 0.490 | 4.513 | 4.14E-05 | 6.72E-04 | S1_D2 - S2_D1 | BA-9_L - BA-46_L |
| Monolingual | 0.528 | 0.587 | 4.587 | 3.23E-05 | 5.61E-04 | S1_D2 - S2_D3 | BA-9_L - BA-10_L |
| Monolingual | 0.660 | 0.792 | 5.730 | 6.47E-07 | 2.52E-05 | S1_D2 - S3_D2 | BA-9_L - BA-9_L |
| Monolingual | 0.673 | 0.816 | 6.811 | 1.43E-08 | 2.24E-06 | S2_D1 - S2_D3 | BA-46_L - BA-10_L |
| Monolingual | 0.571 | 0.649 | 6.409 | 5.93E-08 | 5.04E-06 | S2_D1 - S3_D2 | BA-46_L - BA-9_L |
| Monolingual | 0.658 | 0.790 | 6.269 | 9.72E-08 | 5.83E-06 | S2_D1 - S3_D3 | BA-46_L - BA-10_L |
| Monolingual | 0.667 | 0.806 | 7.417 | 1.69E-09 | 4.40E-07 | S2_D1 - S6_D6 | BA-46_L - BA-10_R |
| Monolingual | 0.577 | 0.659 | 6.323 | 8.03E-08 | 5.22E-06 | S2_D1 - S8_D6 | BA-46_L - BA-46_R |
| Monolingual | 0.561 | 0.635 | 6.338 | 7.63E-08 | 5.22E-06 | S2_D1 - S8_D7 | BA-46_L - BA-46_R |
| Monolingual | 0.560 | 0.633 | 6.229 | 1.12E-07 | 6.20E-06 | S2_D3 - S3_D2 | BA-10_L - BA-9_L |
| Monolingual | 0.606 | 0.703 | 5.891 | 3.68E-07 | 1.79E-05 | S2_D3 - S3_D3 | BA-10_L - BA-10_L |
| Monolingual | 0.630 | 0.741 | 6.674 | 2.32E-08 | 3.02E-06 | S2_D3 - S8_D6 | BA-10_L - BA-46_R |
| Monolingual | 0.445 | 0.478 | 4.592 | 3.18E-05 | 5.61E-04 | S2_D3 - S8_D7 | BA-10_L - BA-46_R |
| Monolingual | 0.664 | 0.800 | 8.056 | 1.81E-10 | 7.07E-08 | S3_D2 - S3_D3 | BA-9_L - BA-10_L |
| Monolingual | 0.542 | 0.608 | 4.844 | 1.37E-05 | 2.86E-04 | S3_D2 - S3_D4 | BA-9_L - BA-9_L |
| Monolingual | 0.477 | 0.519 | 5.415 | 1.94E-06 | 5.59E-05 | S3_D2 - S8_D6 | BA-9_L - BA-46_R |
| Monolingual | 0.447 | 0.481 | 4.554 | 3.61E-05 | 6.01E-04 | S3_D3 - S4_D4 | BA-10_L - BA-8_R |
| Monolingual | 0.474 | 0.515 | 4.553 | 3.62E-05 | 6.01E-04 | S3_D3 - S6_D4 | BA-10_L - BA-9_R |
| Monolingual | 0.620 | 0.725 | 5.590 | 1.05E-06 | 3.43E-05 | S3_D3 - S6_D6 | BA-10_L - BA-10_R |
| Monolingual | 0.496 | 0.544 | 4.922 | 1.05E-05 | 2.34E-04 | S3_D3 - S8_D7 | BA-10_L - BA-46_R |
| Monolingual | 0.451 | 0.486 | 5.008 | 7.85E-06 | 1.86E-04 | S3_D4 - S6_D5 | BA-9_L - BA-9_R |
| Monolingual | 0.537 | 0.601 | 5.334 | 2.57E-06 | 6.90E-05 | S4_D2 - S4_D5 | BA-9_L - BA-8_R |
| Monolingual | 0.587 | 0.674 | 4.374 | 6.55E-05 | 9.64E-04 | S4_D4 - S6_D4 | BA-8_R - BA-9_R |
| Monolingual | 0.551 | 0.620 | 5.663 | 8.18E-07 | 2.90E-05 | S5_D6 - S6_D4 | BA-10_R - BA-9_R |
| Monolingual | 0.683 | 0.836 | 5.866 | 4.02E-07 | 1.84E-05 | S5_D6 - S8_D6 | BA-10_R - BA-46_R |
| Monolingual | 0.618 | 0.721 | 5.534 | 1.28E-06 | 4.00E-05 | S6_D4 - S6_D5 | BA-9_R - BA-9_R |
| Monolingual | 0.480 | 0.523 | 4.399 | 6.02E-05 | 9.03E-04 | S6_D4 - S7_D5 | BA-9_R - BA-9_R |
| Monolingual | 0.619 | 0.724 | 5.464 | 1.63E-06 | 4.90E-05 | S6_D5 - S7_D5 | BA-9_R - BA-9_R |
| Monolingual | 0.551 | 0.620 | 4.400 | 6.01E-05 | 9.03E-04 | S6_D6 - S8_D6 | BA-10_R - BA-46_R |
| Monolingual | 0.682 | 0.834 | 6.385 | 6.47E-08 | 5.04E-06 | S6_D6 - S8_D7 | BA-10_R - BA-46_R |
| Monolingual | 0.551 | 0.620 | 5.293 | 2.95E-06 | 7.66E-05 | S8_D6 - S8_D7 | BA-46_R - BA-46_R |
| *Note.* BA = Brodmann Area; S = Source; D = Detector; L = Left Hemisphere; R = Right Hemisphere; All correlations are significant at an FDR corrected *q-value* of .001. | | | | | | | |

**SI Table 5**

*Significant Correlations for Monolingual Preschoolers – Incongruent Trials*

| Group | *R* | *Z* | *t* | *p-value* | *q-value* | Channel Pairings | ROI Pairings |
| --- | --- | --- | --- | --- | --- | --- | --- |
| Monolingual | 0.489 | 0.534 | 4.476 | 4.67E-05 | 6.08E-04 | S1_D1 - S2_D1 | BA-45_L - BA-46_L |
| Monolingual | 0.467 | 0.506 | 4.629 | 2.81E-05 | 4.39E-04 | S1_D1 - S2_D3 | BA-45_L - BA-10_L |
| Monolingual | 0.449 | 0.484 | 4.692 | 2.29E-05 | 3.79E-04 | S1_D1 - S5_D4 | BA-45_L - BA-10_R |
| Monolingual | 0.418 | 0.446 | 4.333 | 7.47E-05 | 8.02E-04 | S1_D1 - S5_D6 | BA-45_L - BA-10_R |
| Monolingual | 0.488 | 0.533 | 4.814 | 1.51E-05 | 2.88E-04 | S1_D1 - S6_D5 | BA-45_L - BA-9_R |
| Monolingual | 0.420 | 0.448 | 4.723 | 2.05E-05 | 3.55E-04 | S1_D1 - S7_D5 | BA-45_L - BA-9_R |
| Monolingual | 0.478 | 0.521 | 4.585 | 3.26E-05 | 4.54E-04 | S1_D1 - S7_D7 | BA-45_L - BA-45_R |
| Monolingual | 0.461 | 0.499 | 4.905 | 1.11E-05 | 2.48E-04 | S1_D1 - S8_D6 | BA-45_L - BA-46_R |
| Monolingual | 0.436 | 0.467 | 4.303 | 8.23E-05 | 8.45E-04 | S1_D2 - S2_D1 | BA-9_L - BA-46_L |
| Monolingual | 0.579 | 0.661 | 4.784 | 1.67E-05 | 3.11E-04 | S1_D2 - S3_D2 | BA-9_L - BA-9_L |
| Monolingual | 0.492 | 0.538 | 5.291 | 2.98E-06 | 9.11E-05 | S1_D2 - S7_D5 | BA-9_L - BA-9_R |
| Monolingual | 0.592 | 0.681 | 5.688 | 7.49E-07 | 3.65E-05 | S2_D1 - S2_D3 | BA-46_L - BA-10_L |
| Monolingual | 0.496 | 0.544 | 5.368 | 2.28E-06 | 7.56E-05 | S2_D1 - S3_D2 | BA-46_L - BA-9_L |
| Monolingual | 0.603 | 0.699 | 5.544 | 1.24E-06 | 4.82E-05 | S2_D1 - S3_D3 | BA-46_L - BA-10_L |
| Monolingual | 0.678 | 0.826 | 7.602 | 8.83E-10 | 2.85E-07 | S2_D1 - S6_D6 | BA-46_L - BA-10_R |
| Monolingual | 0.499 | 0.548 | 5.258 | 3.33E-06 | 9.62E-05 | S2_D1 - S8_D6 | BA-46_L - BA-46_R |
| Monolingual | 0.567 | 0.643 | 6.420 | 5.71E-08 | 4.95E-06 | S2_D1 - S8_D7 | BA-46_L - BA-46_R |
| Monolingual | 0.515 | 0.569 | 5.604 | 1.00E-06 | 4.35E-05 | S2_D3 - S3_D2 | BA-10_L - BA-9_L |
| Monolingual | 0.652 | 0.779 | 6.531 | 3.85E-08 | 3.75E-06 | S2_D3 - S3_D3 | BA-10_L - BA-10_L |
| Monolingual | 0.379 | 0.399 | 5.014 | 7.67E-06 | 1.81E-04 | S2_D3 - S3_D4 | BA-10_L - BA-9_L |
| Monolingual | 0.471 | 0.511 | 5.362 | 2.33E-06 | 7.56E-05 | S2_D3 - S4_D4 | BA-10_L - BA-8_R |
| Monolingual | 0.431 | 0.461 | 4.341 | 7.28E-05 | 8.00E-04 | S2_D3 - S5_D3 | BA-10_L - BA-10_L |
| Monolingual | 0.443 | 0.476 | 4.422 | 5.59E-05 | 6.88E-04 | S2_D3 - S5_D4 | BA-10_L - BA-10_R |
| Monolingual | 0.526 | 0.584 | 5.080 | 6.13E-06 | 1.54E-04 | S2_D3 - S6_D4 | BA-10_L - BA-9_R |
| Monolingual | 0.471 | 0.511 | 4.376 | 6.50E-05 | 7.35E-04 | S2_D3 - S6_D5 | BA-10_L - BA-9_R |
| Monolingual | 0.422 | 0.450 | 4.312 | 8.00E-05 | 8.32E-04 | S2_D3 - S7_D7 | BA-10_L - BA-45_R |
| Monolingual | 0.606 | 0.703 | 6.328 | 7.88E-08 | 6.15E-06 | S2_D3 - S8_D6 | BA-10_L - BA-46_R |
| Monolingual | 0.612 | 0.711 | 7.166 | 4.10E-09 | 7.99E-07 | S3_D2 - S3_D3 | BA-9_L - BA-10_L |
| Monolingual | 0.519 | 0.575 | 4.581 | 3.30E-05 | 4.54E-04 | S3_D2 - S3_D4 | BA-9_L - BA-9_L |
| Monolingual | 0.586 | 0.672 | 5.114 | 5.46E-06 | 1.42E-04 | S3_D2 - S4_D4 | BA-9_L - BA-8_R |
| Monolingual | 0.381 | 0.402 | 4.332 | 7.50E-05 | 8.02E-04 | S3_D2 - S5_D6 | BA-9_L - BA-10_R |
| Monolingual | 0.543 | 0.608 | 4.814 | 1.51E-05 | 2.88E-04 | S3_D2 - S6_D5 | BA-9_L - BA-9_R |
| Monolingual | 0.421 | 0.449 | 4.347 | 7.13E-05 | 7.95E-04 | S3_D2 - S7_D5 | BA-9_L - BA-9_R |
| Monolingual | 0.481 | 0.524 | 5.465 | 1.63E-06 | 6.05E-05 | S3_D2 - S8_D6 | BA-9_L - BA-46_R |
| Monolingual | 0.461 | 0.498 | 4.718 | 2.09E-05 | 3.55E-04 | S3_D3 - S4_D4 | BA-10_L - BA-8_R |
| Monolingual | 0.486 | 0.531 | 4.585 | 3.26E-05 | 4.54E-04 | S3_D3 - S5_D6 | BA-10_L - BA-10_R |
| Monolingual | 0.576 | 0.657 | 5.069 | 6.36E-06 | 1.55E-04 | S3_D3 - S6_D6 | BA-10_L - BA-10_R |
| Monolingual | 0.549 | 0.617 | 5.285 | 3.04E-06 | 9.11E-05 | S3_D3 - S8_D6 | BA-10_L - BA-46_R |
| Monolingual | 0.491 | 0.537 | 4.859 | 1.30E-05 | 2.67E-04 | S3_D3 - S8_D7 | BA-10_L - BA-46_R |
| Monolingual | 0.525 | 0.584 | 4.376 | 6.50E-05 | 7.35E-04 | S3_D4 - S5_D3 | BA-9_L - BA-10_L |
| Monolingual | 0.431 | 0.461 | 4.745 | 1.91E-05 | 3.47E-04 | S3_D4 - S6_D5 | BA-9_L - BA-9_R |
| Monolingual | 0.372 | 0.391 | 4.383 | 6.35E-05 | 7.35E-04 | S3_D4 - S8_D6 | BA-9_L - BA-46_R |
| Monolingual | 0.691 | 0.849 | 7.541 | 1.10E-09 | 2.85E-07 | S4_D2 - S4_D5 | BA-9_L - BA-8_R |
| Monolingual | 0.486 | 0.531 | 4.246 | 9.91E-05 | 9.52E-04 | S4_D2 - S6_D4 | BA-9_L - BA-9_R |
| Monolingual | 0.530 | 0.590 | 4.591 | 3.20E-05 | 4.54E-04 | S4_D2 - S7_D5 | BA-9_L - BA-9_R |
| Monolingual | 0.574 | 0.653 | 4.239 | 1.01E-04 | 9.53E-04 | S4_D4 - S6_D4 | BA-8_R - BA-9_R |
| Monolingual | 0.453 | 0.488 | 4.271 | 9.13E-05 | 9.13E-04 | S4_D4 - S6_D5 | BA-8_R - BA-9_R |
| Monolingual | 0.518 | 0.574 | 5.182 | 4.33E-06 | 1.17E-04 | S5_D3 - S5_D6 | BA-10_L - BA-10_R |
| Monolingual | 0.471 | 0.511 | 4.860 | 1.30E-05 | 2.67E-04 | S5_D3 - S6_D4 | BA-10_L - BA-9_R |
| Monolingual | 0.426 | 0.454 | 4.601 | 3.09E-05 | 4.54E-04 | S5_D3 - S8_D6 | BA-10_L - BA-46_R |
| Monolingual | 0.522 | 0.579 | 4.243 | 1.00E-04 | 9.52E-04 | S5_D4 - S6_D6 | BA-10_R - BA-10_R |
| Monolingual | 0.487 | 0.532 | 4.580 | 3.32E-05 | 4.54E-04 | S5_D4 - S8_D6 | BA-10_R - BA-46_R |
| Monolingual | 0.559 | 0.631 | 5.761 | 5.80E-07 | 3.01E-05 | S5_D6 - S6_D4 | BA-10_R - BA-9_R |
| Monolingual | 0.598 | 0.691 | 4.848 | 1.35E-05 | 2.70E-04 | S5_D6 - S8_D6 | BA-10_R - BA-46_R |
| Monolingual | 0.451 | 0.486 | 4.438 | 5.30E-05 | 6.78E-04 | S5_D6 - S8_D7 | BA-10_R - BA-46_R |
| Monolingual | 0.621 | 0.727 | 5.582 | 1.08E-06 | 4.45E-05 | S6_D4 - S6_D5 | BA-9_R - BA-9_R |
| Monolingual | 0.518 | 0.574 | 4.718 | 2.09E-05 | 3.55E-04 | S6_D4 - S8_D6 | BA-9_R - BA-46_R |
| Monolingual | 0.571 | 0.649 | 4.896 | 1.15E-05 | 2.48E-04 | S6_D5 - S7_D5 | BA-9_R - BA-9_R |
| Monolingual | 0.582 | 0.666 | 4.671 | 2.45E-05 | 3.90E-04 | S6_D5 - S8_D6 | BA-9_R - BA-46_R |
| Monolingual | 0.543 | 0.609 | 4.318 | 7.86E-05 | 8.28E-04 | S6_D6 - S8_D6 | BA-10_R - BA-46_R |
| Monolingual | 0.675 | 0.820 | 6.277 | 9.47E-08 | 6.71E-06 | S6_D6 - S8_D7 | BA-10_R - BA-46_R |
| Monolingual | 0.478 | 0.520 | 4.287 | 8.67E-05 | 8.79E-04 | S7_D5 - S7_D7 | BA-9_R - BA-45_R |
| Monolingual | 0.608 | 0.705 | 6.021 | 2.33E-07 | 1.30E-05 | S8_D6 - S8_D7 | BA-46_R - BA-46_R |
| *Note.* BA = Brodmann Area; S = Source; D = Detector; L = Left Hemisphere; R = Right Hemisphere; All correlations are significant at an FDR corrected *q-value* of .001. | | | | | | | |
