# Supplementary material for: Examining the role of early bilingualism on interference suppression and prefrontal connectivity
Source: Front Integr Neurosci. 2025 Dec 17;19:1591250. doi: 10.3389/fnint.2025.1591250 (PMC12753874; doi:10.3389/fnint.2025.1591250)
Supplement: Supplementary file 4 [file Table_4.docx]

**The following results were significant at an uncorrected *p-value* < .05 and thus should be interpreted with caution. They, however, may be representative of trends of which the current study is underpowered to examine fully and thus are important for future research in this area.**

For congruent trials, bilingual preschoolers showed an increase in HbO activation for channel S2-D3*,* *β* = 7.70, *SE* = 3.48, *t*(46) = 2.21, *p =* .03*.* Monolingual preschoolers showed increase in HbO activation in channels S1-D2, *β* = 8.73, *SE* = 3.17, *t*(46) = 2.76, *p =* .01*,* S4-D5, *β* = 10.20, *SE* = 3.18, *t*(46) = 3.21, *p <* .01, and S7-D5, *β* = 8.02, *SE* = 2.73, *t*(46) = 2.94, *p <* .01, and a significant decrease in HbO activation in channel S4-D2, *β* = -5.62, *SE* = 2.61, *t*(46) = -2.16, *p =* .04.

For incongruent trials, bilingual preschoolers had no increases in HbO activation; however, there were significant decreases in HbO activation for this group in channels S1-D1*,* *β* = -11.14, *SE* = 3.64, *t*(46) = -3.06, *p <* .01, and S3-D2*,* *β* = -12.21, *SE* = 4.03, *t*(46) = -3.03, *p <* .01. Monolingual preschoolers showed increases in HbO activation in channels S1-D2*,* *β* = 12.49, *SE* = 3.46, *t*(46) = 3.61, *p <* .001*,* S4-D5*,* *β* = 16.25, *SE* = 3.39, *t*(46) = 4.79, *p <* .001*,* S5-D6*,* *β* = 7.05, *SE* = 3.50, *t*(46) = 2.01, *p <* .05, and S7-D5*,* *β* = 6.96, *SE* = 2.84, *t*(46) = 2.45, *p =* .02.

When comparing groups by trial types, the monolingual preschoolers showed greater HbO activation during congruent trials than the bilingual preschoolers in channels S1-D2*,* *β* = 8.73, *SE* = 3.17, *t*(46) = 2.76, *p <* .01, S4-D5*,* *β* = 10.20, *SE* = 3.18, *t*(46) = 3.21, *p <* .01, and S7-D5*,* *β* = 8.02, *SE* = 2.73, *t*(46) = 2.94, *p <* .01. Monolingual preschoolers showed significantly decreased activation than the bilingual preschoolers in channel S4-D2*,* *β* = -5.62, *SE* = 2.61, *t*(46) = -2.16, *p <* .05 for congruent trials. For incongruent trials, the monolingual preschoolers again showed significantly greater HbO activation during than the bilingual preschoolers in channels S1-D2*,* *β* = 12.49, *SE* = 3.46, *t*(46) = 3.61, *p <* .001, S4-D5*,* *β* = 16.25, *SE* = 3.39, *t*(46) = 4.79, *p <* .001, S5-D6*,* *β* = 7.05, *SE* = 3.50, *t*(46) = 2.01, *p <* .05, and S7-D5*,* *β* = 6.96, *SE* = 2.84, *t*(46) = 2.45, *p =* .02.
